# Robot-assisted Nipple-sparing Mastectomy with Immediate Breast Reconstruction: An Initial Experience

**DOI:** 10.1038/s41598-019-51744-2

**Published:** 2019-10-30

**Authors:** Hyung Seok Park, Jeea Lee, Dong Won Lee, Seung Yong Song, Dae Hyun Lew, Seung Il Kim, Young Up Cho

**Affiliations:** 1 Department of Surgery, Yonsei University College of Medicine, 50-1, Yonsei-ro, Seodaemun-gu, Seoul, 03722 Republic of Korea; 2 Department of Plastic and Reconstruction Surgery, Yonsei University College of Medicine, Seoul, Korea

**Keywords:** Breast cancer, Breast cancer, Surgical oncology

## Abstract

Seeking smaller and indistinct incisions, physicians have attempted endoscopic breast surgery in breast cancer patients. Unfortunately, there are some limitations in the range of movement and visualization of the operation field. Potentially addressing these limitations, we investigated the outcomes of gas and gasless robot-assisted nipple-sparing mastectomy (RANSM) with immediate breast reconstruction (IBR). Ten patients underwent 12 RANSM with IBR between November 2016 and April 2018. Patients with tumors measuring >5 cm in diameter, tumor invasion of the skin or nipple-areolar complex, proven metastatic lymph nodes, or planned radiotherapy were excluded. Age, breast weight, diagnosis, tumor size, hormone receptor status, and operation time were retrospectively collected. Postoperative outcomes including postoperative complications and final margin status of resected were analyzed. The median total operation time and console time were 351 min (267–480 min) and 51 min (18–143 min), respectively. The learning curve presented as a cumulative sum graph showed that the console time decreased and then stabilized at the eighth case. There was no open conversion or major postoperative complication. One patient had self-resolved partial nipple ischemia, and two patients experienced partial skin ischemia. We deemed that RANSM with IBR is safe and feasible for early breast cancer, benign disease of the breast, and *BRCA 1/2* mutation carriers. RANSM is an advanced surgical method with a short learning curve.

## Introduction

Nipple-sparing mastectomy (NSM) is popular secondary to the excellent cosmetic effects achieved in appropriately selected patients^1^. However, a conspicuous scar on the breast dome and a change in the shape of the breast are unavoidable. An inframammary fold or lateral or periareolar incision ensures an inconspicuous scar; however, removal of adequate quantities of breast tissue is technically challenging^2^. The risk of nipple necrosis associated with a periareolar incision is higher than that with a non-periareolar incision^3^. Aesthetically, endoscopic breast surgery is expected to achieve complete cancer clearance with preservation of the patient’s body image^4^. However, restricted maneuverability (because of inflexible endoscopic equipment) and inadequate operative field visualization (because of 2-dimensional cameras) are limitations of this technique^4,5^.

Robotic surgery is widely used across various fields since its introduction in 1985^6^ because of its several incomparable advantages. High-resolution, 10-fold image magnification, and 3-dimensional optics enable accurate visualization and differentiation of fine structures including intercostal perforators and lymphatics^7^. The sophisticated and intuitive motion of robotic arms allows microscale manipulation, and surgeons can perform delicate tasks accurately even in a limited operative field. Thus, robotic surgery scores over endoscopic surgery and is widely used for several intracorporeal procedures. Toesca *et al*. first introduced robotic breast surgery in 2015 after which this technique was attempted globally^5,8^. Only a few studies have evaluated the feasibility and safety of robot-assisted nipple-sparing mastectomy (RANSM) with immediate breast reconstruction (IBR) to treat breast cancer. Not many studies have analyzed the learning curve of RANSM. We investigated the outcome of RANSM with IBR and analyzed the learning curve for this procedure.

## Patients and Methods

### Cadaveric study

**Figure 1:**
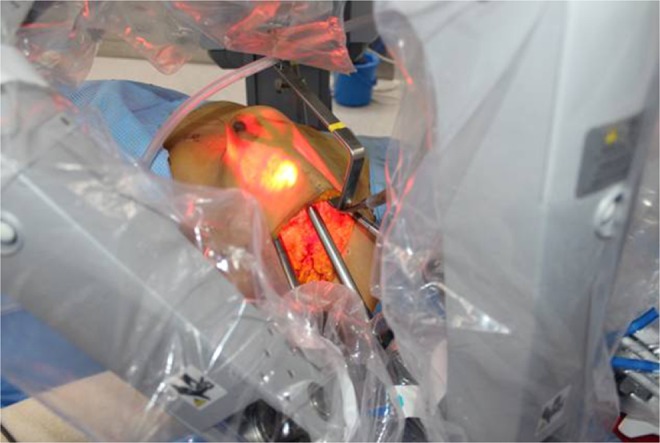
The cadaveric study of robot-assisted nipple-sparing mastectomy with immediate breast reconstruction.

Prior to evaluating the feasibility and safety of RANSM with IBR in humans, RANSM with a latissimus dorsi flap and/or implant insertion was performed on cadavers between December 2013 and November 2014 (Fig. 1). The sources of cadavers were body donation programs or unclaimed bodies provided by the Severance robot and MIS center^9,10^. Six breasts in 3 cadavers were used for the initial RANSM with IBR, and feasibility and safety of the procedure were evaluated. The cadaveric study was performed by 2 breast surgeons and 2 plastic surgeons. A Chung’s or modified Chung’s retractor for endoscopic and robotic surgeries was utilized during the gasless technique. The detailed procedure of a gasless RANSM has been described in a previous case report^11^. During the first attempted cadaveric RANSM, the breast surgeons could not identify the anatomical borders of the breast. Thus, indigo carmine was injected into the borders of the breast parenchyma to identify the anatomical borders of the breast. After injection of the dye, the anatomical borders of the breast could be identified within the working space without help from assistants during the second and third cadaveric studies. Barring this aforementioned technical issue, no significant failure or safety issue occurred during cadaveric RANSM with IBR. In addition to the cadaveric study, the surgeons also participated in the essential robot-training programs conducted by the Severance Robot & Minimally Invasive Surgery center to gain experience in basic and advanced robotic surgical skills.

### Patient selection

Between November 2016 and April 2018, 10 patients underwent RANSM with IBR at a single center. This study was approved by the Institutional Review Board of Severance Hospital including waiver of consent for the current study because the study involved only a retrospective chart review of anonymous patients.

A single breast surgeon and 2 plastic surgeons performed 12 RANSM with IBR (2 patients underwent bilateral procedures). Selected patients underwent physical examination, mammogram, breast ultrasonography and magnetic resonance imaging. Chest or abdominopelvic computed tomography, abdominal ultrasonography, or whole body bone scan were performed to assess metastasis. Patients with tumors measuring >5 cm in diameter, those with skin or nipple-areolar complex (NAC) tumor invasion, those showing metastatic lymph nodes, or those scheduled for radiotherapy were excluded. We recorded age, body mass index, breast weight, diagnosis, tumor size and grade, number of metastatic lymph nodes, hormone receptor status, and type of adjuvant therapy. Postoperative complications and final margin status of resected specimens were evaluated to investigate postoperative outcomes. The operation time was measured as the interval between the creation of the skin incision and the end of the reconstructive surgery. The console time was defined as the time spent by a surgeon to operate the console for mastectomy.

### Surgical technique

The procedure was performed in the first 10 patients using a gasless technique with self-retractors used for robotic and endoscopic surgery. Briefly, a patient was placed in the supine position using a shoulder pad. The ipsilateral arm was straightened with internal rotation and abduction and fixed to the head. Indigo carmine was injected around the NAC to detect sentinel lymph nodes preoperatively. A 3.5–6 cm sized longitudinal skin incision was made in the anterior axillary line below the axillary fossa. Sentinel lymph node biopsy was performed manually without robotic assistance, and the subcutaneous skin flap was dissected toward the NAC using monopolar electrocautery. After an adequate working space was obtained, the retroareolar ductal tissue was resected for frozen section examination to evaluate tumor involvement. Before docking the da Vinci Xi^®^ or Si^®^ Surgical System (Intuitive Surgical Inc., California, US), indigo carmine was injected into the borders of the breast to delineate the anatomical boundaries of the breast.

In the gasless method, the retractor was hung to lift the skin flap, and the robot was docked on the contralateral side of the patient. Dissection was performed using fenestrated bipolar forceps on the left side and a permanent cautery spatula on the right under visual guidance of a dual-channel 30° down endoscope of the robotic system^11^.

After dissecting the subcutaneous flap, a Chung’s or modified Chung’s retractor was repositioned over the pectoralis major fascia and drawn up to expose the posterior aspect of the breast. The breast tissue was detached from the fascia and removed through the 3.5–6 cm axillary skin incision.

RANSM with IBR using gas insufflation was performed in the 2 most recent cases as described in a previous study^7^. In brief, carbon dioxide (CO2) gas was insufflated through a single port (Lapsingle^®^, Sejong Medical Inc., Korea). The retroareolar ductal tissue was resected for frozen section examination under robotic vision. Dissection of the skin flap was performed using the ProGrasp^TM^ forceps (Intuitive Surgical, Sunnyvale, USA) on the left side and the Hot Shears^TM^ monopolar curved scissors (Intuitive Surgical, Sunnyvale, USA) on the right.

Assistants guided the surgeon by providing verbal information/guidance regarding the location of the instrument’s tip and the flap thickness. After the entire breast tissue was removed, the subcutaneous skin flap was inspected for bleeding and thickness, and the IBR was performed by the plastic surgeons.

### Statistical analysis

This study used 3 case moving average curves (MAC) and cumulative sum (CUSUM) analysis to identify the learning curve for RANSM except 2 gas methods of RANSM because different technique can affect the result of the learning curve. Previous studies have reported the utility of MAC and CUSUM in analyzing the learning curves^12–14^. The slope of the CUSUM graph represents the trend of learning performance, and the plateau represents the proficiency acquired.

The SPSS software, version 23 (SPSS Inc., Chicago, IL) was used for statistical analysis. The polynomial curves of the MAC and CUSUM diagram were obtained using the Trendline function of Microsoft Excel 2010, and we subsequently determined the curve gradients.

## Results

### Clinical and pathological characteristics

The median age of patients was 46 years (29–51 years), and the median weight of the resected breast specimens was 225.5 g (150–436 g). Ten mastectomies were performed to treat breast cancer—9 cases (75%) presented with invasive ductal carcinoma and 1 case (8.3%) with ductal carcinoma *in situ* (DCIS). The patient with DCIS underwent a bilateral procedure, and a bilateral procedure was also performed in a patient with interstitial mastitis secondary to a foreign body injection. The median tumor size was 1.35 cm (0.5–4.2 cm). Sentinel lymph node biopsies were performed during all operations except in 1 patient with interstitial mastitis. Axillary dissection without robotic assistance was performed in a patient showing a positive frozen section biopsy result. One patient with a metastatic lymph node that was not identified by the frozen section underwent postoperative radiation therapy. All patients with breast cancer showed estrogen receptor positivity and received hormonal therapy. Human epidermal growth factor receptor 2 was overexpressed in 3 patients, and 1 of them received targeted therapy. Left breast surgeries were performed in 5 and right breast surgeries in 7 cases. Direct-to-implant breast reconstruction was performed in 3 and reconstruction using tissue expanders in 9 cases.

Adjuvant chemotherapy was administered in 4 and radiotherapy in 2 patients.

All frozen sections were examined, and histopathological changes were detected in 2 patients. One patient with a superficial focal abutting of the skin margin underwent radiotherapy. The other patient showed ductal carcinoma of the NAC and underwent excision of the NAC (Tables 1 and 2).

**Table 1:** General characteristics of the study population.

	RANSM with IBR (n = 12)
Age (years)	46 (29–51)
BMI (kg/m^2^)	20.8 (18.59–23.93)
Breast weight (g)	225.5 (150–436)
**Diagnosis**	
Benign	2 (16.7)
DCIS	1 (8.3)
IDC	9 (75)
Tumor size (cm) (n = 10)*	2.3 (0.5–4.2)
No. of metastatic lymph nodes (n = 10)*	
0	8 (80)
1	2 (20)
Histopathological grade (n = 10)*	
1	0 (0)
2	6 (60)
3	4 (40)
Estrogen receptor status (n = 10)*	
Negative	0 (0)
Positive	10 (100)
Progesterone receptor status (n = 10)*	
Negative	1 (10)
Positive	9 (90)
HER2 status (n = 10)*	
Negative	7 (70)
Positive	3 (30)
Ki 67 (n = 10)*	
Low (<14%)	5 (50)
High (≥14%)	5 (50)
Adjuvant chemotherapy (n = 10)*	
No	6 (60)
Yes	4 (40)
Radiotherapy (n = 10)*	
No	8 (80)
Yes	2 (20)
Hormonal therapy (n = 10)*	
No	0 (0)
Yes	10 (100)
Targeted therapy (n = 10)*	
No	9 (90)
Yes	1 (10)

Values are represented as median (minimum–maximum) or N (percentage).

*2 cases showed a benign presentation.

BMI: body mass index, DCIS: ductal carcinoma *in situ*, HER: human epidermal growth factor receptor, IBR: immediate breast reconstruction, IDC: invasive ductal carcinoma, RANSM: robot-assisted nipple-sparing mastectomy.

**Table 2:** Surgical methods and postoperative outcomes.

	RANSM with IBR (n = 12)
**Operated site**	
Left	5 (41.7)
Right	7 (58.3)
**Reconstruction**	
Tissue expander insertion	9 (75)
Direct-to-implant	3 (25)
**Gas insufflation**	
No	10 (83.3)
Yes	2 (16.7)
SLNB (n = 10)*	
No	0 (0)
Yes	10 (100)
ALND (n = 10)*	
No	9 (90)
Yes	1 (10)
Length of hospitalization (days)	11 (9–13)
Total operation time (min)	351 (267–480)
Console time (min)	51 (18–143)
**Margin status**	
Negative	10 (83.3)
Positive	2 (16.7)
**Complication**	
None	9 (75)
Skin ischemia	2 (16.7)
Nipple ischemia	1 (8.3)

Values are represented as median (minimum–maximum) or N (percentage).

*2 cases showed a benign presentation.

ALND: axillary lymph node dissection, IBR: immediate breast reconstruction, RANSM: robot-assisted nipple-sparing mastectomy, SLNB: sentinel lymph node biopsy.

### Operation time

**Figure 2:**
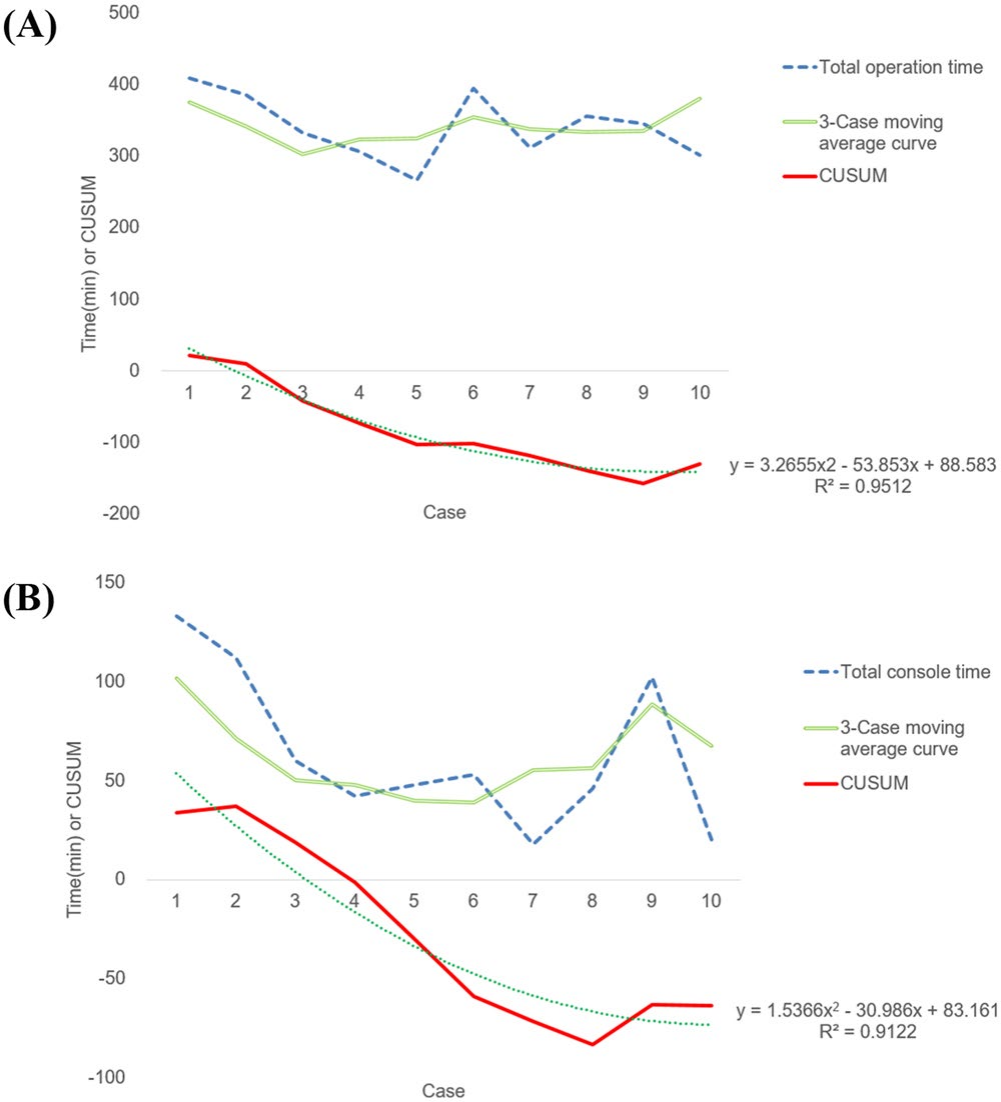
The learning curve of the robot-assisted nipple-sparing mastectomy with immediate breast reconstruction. (**A**) The learning curve of the total operation time. (**B**) The learning curve of the console time. CUSUM: cumulative sum.

The median length of hospitalization was 11 days (9–13 days). The median operation time was 351 min (267–480 min). The median console time was 51 min (18–143 min) (Table 2). The first 5 patients who underwent the operation showed a shorter total operation and console time. However, from the sixth case onwards, we observed fluctuations in the operation times. The MAC graph of the 3 cases representing the total operation time and the console time also decreased until the fifth case and increased from the sixth case onwards. CUSUM analysis showed that the total operation time reduced until the ninth case and increased thereafter (Fig. 2A). The CUSUM graph of the console time decreased until the eighth case and stabilized thereafter (Fig. 2B).

### Postoperative complications

Conversion to open surgery and mortality were not reported. Major postoperative complications including hematoma, infection, and total nipple or skin necrosis did not occur. Self-limited partial nipple ischemia occurred in 1 and partial skin ischemia in 2 patients (Table 2).

## Discussion

This study showed that RANSM with IBR is safe and feasible for patients with early breast cancer. With improved disease-free and overall survival rates, patients with early breast cancer are increasingly concerned regarding postoperative cosmetic effects and overall quality of life^5^. Therefore, newer surgical methods that ensure oncologic safety without compromising body image are required. We introduced robotic breast surgery to achieve these goals. The new surgical method facilitates RANSM with IBR for patients with early breast cancer via a small axillary incision. No major postoperative complications including total nipple necrosis, wound infection, and implant removal were observed. No negative effects on cosmetic outcomes and quality of life were identified. Previous studies have reported similar results of RANSM with IBR^5,8,15^.

Gasless RANSM with IBR was performed for the first time in our study. The gasless and the CO2-insufflation techniques are both associated with specific advantages and disadvantages. The gasless technique utilizes a self-retractor (Chung’s retractor) to maintain the working space as an alternative to CO2 gas insufflation^16^. A disadvantage of the CO2-insufflation method is that CO2 gas leakage impairs optimal availability and visualization of the working space, and robotic vision can be affected by the smoke released during electrocauterization. The gasless technique utilizes retractors to maintain the working space; thus, bleeding or air suction associated with this procedure does not significantly impair the availability and visualization of the working space. The smoke from electrocauterization causes lesser technical difficulties (lesser visual disturbances) with the gasless technique thereby facilitating a clearer view of the operative field. Postoperative subcutaneous emphysema and hypercapnia can complicate the gas insufflation technique^5^. Although these complications can be managed with supportive care^15^, the gasless robotic procedure eliminates the risks of CO2 insufflation-related complications. Another benefit of the gasless technique is that the retroareolar ductal tissue can be resected to confirm tumor invasion by performing frozen section examination prior to docking of the robotic system.

A few disadvantages of the gasless technique must be mentioned. Prolonged retraction can cause skin flap ischemia. The length of a single incision required for the gasless technique is longer than that used for the gas-insufflated procedure. In this study, we used an axillary incision measuring 2.5–4 cm for the CO2-insufflation technique compared with an incision measuring approximately 3.5–6 cm for the gasless technique.

Robotic surgery is more ergonomically comfortable than conventional surgery owing to the 3-dimensional optics and intuitive robotic arm motion^6^, and robotic surgery is easier to learn than conventional surgery. As reported by previous studies, learning curves for RANSM are rapidly declining^5,15,17^. In this study, the console time was investigated to evaluate proficiency of surgical skills in robotic surgery. CUSUM analyses of the console time revealed that it was rapidly stabilized at the eighth case. It can be concluded that surgeons can acquire appropriate surgical skills within a relatively short time.

Owing to limited data regarding long-term outcomes, RANSM is performed only in highly selected patients, primarily patients with early breast cancer, healthy carriers showing *BRCA1/2* mutation, and those with benign disease. In this study, nearly all patients demonstrated early breast cancer. Toesca *et al*. enrolled patients with early breast cancer and carriers with *BRCA1/2* mutations^5,7^. Sarfati *et al*. suggested that unaffected carriers with *BRCA1/2* mutation could be good candidates for RANSM with IBR^8,17^. Indications outlined in the previous study were similar to those in our study. In the current study, 1 patient underwent RANSM with IBR because of bilateral interstitial mastitis. A previous study has suggested that NSM with IBR can be used to treat this condition^18^. We reported the first attempt to treat bilateral interstitial mastitis using robotic surgery (Table 3).

**Table 3:** Comparison between previous studies and the current study describing robot-assisted nipple-sparing mastectomy with immediate breast reconstruction.

Study	Type of study	Number of procedures performed (N)	Age (years)	Indications (N, %)	Method of RANSM	Reconstruction method	Breast volume (g)	Open conversion (N, %)	Blistering of skin (N, %)	NAC necrosis (N, %)	Infection or delayed wound healing (N, %)	f/u (months)
Toesca A. *et al*. (2017)	Prospective	29	42 (30–55)	*BRCA* mutation carrier (11, 38%) DICS (9, 31%) Invasive carcinoma (9, 31%)	with gas	expander or DTI	range 200–300	2, 6.9%	2, 6.9%	0	0	8 (1–14)
Sarfati B. *et al*. (2018)	Prospective	63	37 (24–52)	DCIS (1, 1.6%) *BRCA* mutation carrier (62, 98.4%)	with gas	expander or DTI	range 78–330	1, 1.6%	2, 3.2%	0	3, 4.8%	9 (NA)
Lai HW. *et al*. (2018)	Retrospective	23	48.9*	NA	with gas	DTI	284.3*	0	2, 8.6%	partial (3, 13%)	1, 4.3%	6.9*
Park HS *et al*. (Present)	Retrospective	12	46 (29–51)	Benign (2, 16.7%) DCIS (1, 8.3%) IDC (9, 75%)	with or without gas	expander or DTI	range 150–436	0	2, 16.7%	partial (1, 8.3%)	0	8 (5–22)

Values are expressed as median (minimum–maximum) or (N, percentage).

*These are mean values.

DCIS: ductal carcinoma *in situ*, DTI: direct-to-implant, f/u: follow-up, IBR: immediate breast reconstruction, IDC: invasive ductal carcinoma, NA: not available, NAC: nipple-areolar complex, RANSM: robot-assisted nipple-sparing mastectomy.

No major complications were reported among patients who underwent RANSM with IBR. A previous study reported a postoperative complication rate of 10.3%^5^. Skin blistering, partial necrosis of the skin and nipple, and temporary neurapraxia are common postoperative complications of RANSM^5,15,17^. These complications can be managed conservatively as reported by previous studies, which concurs with our results. Toesca *et al*. reported that the conversion rate to open surgery was 6.9% in those who underwent RANSM; however, no patient required conversion to open surgery in our study^5^ (Table 3).

The limitations of this study are as follows: Owing to the small size and retrospective design, the role of a selection bias and uncontrolled confounders cannot be completely excluded. Furthermore, long-term survival outcomes remain unknown because this is our initial experience of RANSM with IBR. RANSM with IBR was performed only in a highly selected group of patients such as those with early breast cancer and benign disease. To overcome these limitations, prospective trials are warranted to evaluate the stability and the cosmetic effects of the surgery. RANSM with IBR has not been standardized because of the insufficient evidence regarding the safety and efficacy of the various methods. Large prospective randomized studies are needed to definitively establish a more accurate and stable surgical procedure.

## Conclusion

RANSM with IBR is a feasible and safe surgical treatment for early breast cancer and benign conditions. Although robotic technology is a relatively recent introduction, RANSM with IBR showed a rapid learning curve.

## Data Availability

The data of the current study are available from the corresponding author on reasonable request.
