# Author Correction: Robot-assisted Nipple-sparing Mastectomy with Immediate Breast Reconstruction: An Initial Experience

**DOI:** 10.1038/s41598-020-64238-3

**Published:** 2020-04-30

**Authors:** Hyung Seok Park, Jeea Lee, Dong Won Lee, Seung Yong Song, Dae Hyun Lew, Seung Il Kim, Young Up Cho

**Affiliations:** 1 Department of Surgery, Yonsei University College of Medicine, 50-1, Yonsei-ro, Seodaemun-gu, Seoul, 03722 Republic of Korea; 2 Department of Plastic and Reconstruction Surgery, Yonsei University College of Medicine, Seoul, Korea

Correction to: *Scientific Reports* 10.1038/s41598-019-51744-2, published online 30 October 2019

In the original version of this Article, Hyung Seok Park and Jeea Lee were omitted as equally contributing authors. This has now been corrected in the PDF and HTML versions of the paper.

